# Sex and the Syndrome: Individual and Population Consistency in Behaviour in Rock Pool Prawn *Palaemon elegans*

**DOI:** 10.1371/journal.pone.0059437

**Published:** 2013-03-15

**Authors:** Ben B. Chapman, Alexander Hegg, Peter Ljungberg

**Affiliations:** Aquatic Ecology Unit, Department of Biology, Lund University, Lund, Skåne, Sweden; CNRS, Université de Bourgogne, France

## Abstract

Animal personality has been widely documented across a range of species. The concept of personality is composed of individual behavioural consistency across time and between situations, and also behavioural trait correlations known as behavioural syndromes. Whilst many studies have now investigated the stability of individual personality traits, few have analysed the stability over time of entire behavioural syndromes. Here we present data from a behavioural study of rock pool prawns. We show that prawns are temporally consistent in a range of behaviours, including activity, exploration and boldness, and also that a behavioural syndrome is evident in this population. We find correlations between many behavioural traits (activity, boldness, shoaling and exploration). In addition, behavioural syndrome structure was consistent over time. Finally, few studies have explicitly studied the role of sex differences in personality traits, behavioural consistency and syndrome structure. We report behavioural differences between male and female prawns but no differences in patterns of consistency. Our study adds to the growing literature on animal personality, and provides evidence showing that syndromes themselves can exhibit temporal consistency.

## Introduction

Individual personality was once thought to be a uniquely human phenomenon [1]. However, a plethora of recent studies now show that the existence of individual personality is taxonomically widespread [2], with data from animals as diverse as non-human primates [3], birds [4] and fish [5], highlighting that in fact many animals show consistency in their behaviour. Perhaps more surprising are studies that show that individual differences in behaviour (often referred to as ‘animal personality’) are also evident in many animals with much more limited cognitive capacities such as insects [6], arachnids [7], molluscs [8] and crustaceans [9]. Evidence supporting the near ubiquity of the personality phenomenon across the animal kingdom has driven a rapid expansion of this research field in recent years, particularly for research investigating the evolutionary basis or ecological consequences of personality variation [10], [11]. Now data shows that behavioural consistency over time and across situations occurs for many traits, for example boldness [12], activity [13], exploration [14], mating behaviour [15], and also under field conditions for ecologically important behaviours such as migratory behaviour [16]. However the concept of personality involves not only behavioural consistency over time or situation, but also the idea that an individual can have a “behavioural type” [17]: i.e. that linkages can exist between functionally different behaviours. These linkages, when conceptualised as correlations at a population level, are known as behavioural syndromes [17]. Examples of behavioural syndromes in nature are population-level positive correlations between boldness, aggression and activity in red ants [18], or aggression and boldness in female spiders [19]. Both consistency and the presence of behavioural syndromes are thought to be different facets characterising animal personality. Both components of personality are becoming increasingly well studied, however only a handful of studies (e.g. Bell & Sih, [20] Adriaenssens & Johnsson [21]) have broached a key and unresolved question that goes to the heart of bridging these two separate properties which together characterise animal personality: to what degree are behavioural syndromes consistent over time? Whilst many single behaviours have been shown to be consistent in many examples, are the trait-linkages also consistent?

The existence of personalities within natural populations of animals is thought to have powerful consequences for ecology and evolution [10], [11], [22], and so understanding the factors that promote such variation is of great interest to biologists. An important source of behavioural variation in animals is driven by sex differences. Many studies have reported differences between males and females in a variety of behavioural traits, for example in guppies *Poecilia reticulata,* males are bolder than females following a simulated predator attack [23]. The role of sex differences in shaping personality variation is an area that has also begun to receive attention in the behavioural sciences (e.g. Schuett & Dall [24], Pruitt & Riechert [25], Ruuskanen & Laaksonen [26]), however data remains relatively scarce. Do males and females have a similar degree of consistency in behaviour? Do patterns of correlation between different behavioural traits differ between sexes? In this paper we address a number of questions about animal personality using the rock pool prawn *Palaemon elegans* as a model organism. We asked (1) is individual prawn behaviour (across a range of traits: activity, boldness, exploration, shoaling tendency) consistent over time; (2) are these behaviours correlated (i.e. do they form a behavioural syndrome); (3) is syndrome structure consistent over time, and finally (4) are there sex differences in behaviour, consistency and syndrome structure between male and female prawns? We predicted that sexes would differ in key behaviours such as boldness and activity due to the divergent requirements of males and females in terms of sexual behaviour, with males being more active, exploratory and bold than females. We had no strong *a priori* predictions about sex differences in consistency or syndrome structure.

## Materials and Methods

### Study System

The rock pool prawn, *P. elegans*, is a widespread species that is common on the Swedish west coast, inhabiting coastal eel grass, brown algae and open sand habitats [27]. It is omnivorous, feeding on a wide range of food sources, including filamentous algae and small crustaceans [27], [28] and is also an important food source for fish such as juvenile cod (*Gadus morhua*) that inhabit and use shallow coastal areas [29]. We collected prawns in June 2010, using sweep nets from three locations, Domsten harbour (N 56° 7′ 1″, E 12° 36′ 11″), Höganäs harbour (N 56° 11′ 56″, E 12° 32′ 46″) and Ålabodarna harbour (N 55° 43′ 39″, E 12° 57′ 27″). The areas were on a continuous stretch of coast with similar habitat. *P. elegans* have a pelagic larval stage lasting three to five weeks and can travel up to 600 km [30] which suggests that there is a considerable exchange of genetic material between these areas. Hence we considered all individuals as part of the same population.

We transported prawns to the laboratory and immediately transferred them to aerated aquaria with 12 h:12 h (Light/Dark) ratio, a water temperature of 21°C and a salinity of 18 ‰, which corresponds to field conditions. Individuals were allowed to acclimate for at least 1 week prior to the onset of behavioural trials to characterise personality. A total of 90 individuals from the pool of collected individuals were selected haphazardly for inclusion in the study. Individuals of varying size classes were selected as males and females can differ in size [31]. They were transferred individually to separate oxygenated (17×14×10 cm) plastic containers. This made it possible to keep track of every individual throughout the experimental period.

### Behavioural Assays

Individuals were each assayed for their behaviour over a period of two weeks. Each prawn was assayed to quantify activity, shoaling tendency, habitat preference/exploration and boldness, in this order. One behaviour was quantified each day, and the trials were repeated to assess consistency on the second week, in the same order as the first week. We adopted a sequential approach to control for differences in recent experience that would have been generated if trials had been randomly ordered (discussed in Bell [32]). Prawns were fed once every week with fish pellets in order to keep hunger level consistent for all experimental individuals. At the end of the trials the individuals were sacrificed using 70% ethanol and analysed to determine sex by presence or absence of the appendix masculina (N males = 46, N females = 34).

#### Activity

Each focal individual was placed in a 33×25×17 cm opaque plastic container (water depth = 8 cm) and left to acclimatise for 3 minutes before the trial began. We then recorded individual movement over a further 3-minute period using a digital video camera. At the end of a trial focal prawns were returned to their home container and 250 ml water was changed to avoid the build up of chemical cues. To calculate individual activity we divided the arena into 12 equally sized square grids (8.3×8.3 cm) and recorded the number of times an individual changed zone during the trial.

#### Shoaling tendency

**Figure 1:**
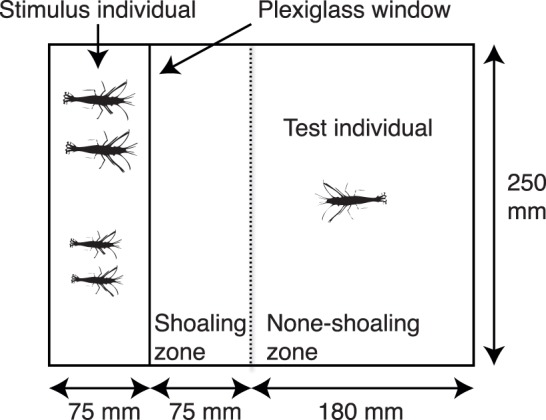
Experimental set-up for the shoaling behaviour trial.

To quantify shoaling tendency we used a standard choice chamber, e.g. Chapman et al. [33]. One experimental arena (33×25×17 cm) was divided into two, separated by a transparent plexiglas window (Fig. 1). In one compartment (7.5×25×17 cm) we placed four stimulus individuals to represent a social group. Small holes had been drilled through the window to permit water to pass through the compartments, thus allowing chemical cues from conspecifics to seep into the focal compartment. We also designated a ‘social zone’ which was 7.5 cm from the social compartment within the focal compartment. Following the introduction of the stimulus shoal, we placed the focal individual in the focal compartment. All individuals were allowed to acclimatise for 3 minutes before the trials begin. Following this, shoaling behaviour was recorded from above using a digital video camera for a further 3-minute period. To quantify individual shoaling tendency we calculated the proportion of time a focal prawn spent in the shoaling zone, following Evans et al. [34]. After every other trial the stimulus individuals were replaced and 250 ml water was changed to avoid the build up of chemical cues in the water.

#### Habitat preference and exploratory tendency

An experimental arena (33×25×17 cm) was divided into two equally sized zones where half of the arena consisted of a synthetic eelgrass (*Zostera* sp.) habitat (500 strands/m^2^), and the remaining half of plain open sand habitat. Previous work has shown that prawns are more exposed to predation in less structured habitats [35]. All sides of the tank were opaque except one, to create a clear transparent plexiglas window to observe prawn habitat use. Following an acclimatisation period of 3 minutes, we ran trials for 7 minutes. Every 30 seconds, the location of the prawn was observed and noted (inside or outside the eelgrass). Here we calculated the proportion of time spent in the eelgrass habitat and also the number of times an individual switched zones, to give us an insight into the exploratory behaviour of prawns (from herein this trait will be referred to as ‘exploration’).

#### Fright response (boldness)

We used a smaller arena, measuring 17×14×10 cm (water depth 6 cm), to assess individual boldness. The bottom of the arena was filled with 1 cm of sand and a small piece of food (unfrozen shrimp, approx. 1 cm^3^) was placed in the center of the arena, attached to a steel wire that was glued to a small rock buried in the sand to anchor the food to one position. The test individual was acclimatised for 1 minute in a transparent cylinder (6 cm in diameter) in the bottom left corner of the arena. Following acclimatisation, the focal individual was allowed to locate the food. Once the test individual started to feed, a stainless steel weight (58.7 g) was dropped into the water to startle the focal prawn. We then measured the time taken to return to feed as a measure of fright response. If the individual had not returned within 5 minutes (300 s) we assigned it a ceiling value of 300 s. Individuals with low scores returned quickly to the food and hence for this trial a low score indicates a bold individual.

### Statistical Methodology

All statistics were carried out in R [36]. Individual consistency was determined using Spearman’s rank correlation tests, as were correlations to investigate behavioural syndrome structure. Sex differences in behaviour were assessed using t-tests where data conformed to assumptions, and Mann-Whitney U tests where parametric assumptions were violated. Taking the correlation coefficients for all pairwise behavioural trait comparisons in week 1 and week 2 allowed us to test for syndrome stability over time. We built matrices of correlation coefficients and tested whether the correlation matrices were related to each other (i.e. consistent over time in behavioural syndrome structure) using a Mantel test. Mantel tests evaluate the correlation between different matrices using a permutation procedure. We also used this approach to compare the syndrome structure of males and females (using syndrome data from week 1 in this analysis). Throughout we correct for multiple testing using q values to control the false discovery rate for the analysis of correlated behaviours [37]. The q value of a test measures the proportion of false positives incurred (false discovery rate) when that particular test is statistically significant.

### Ethics Statement

No specific permits were required for the described field studies. The sampling location is not privately-owned or protected in any way and the study did not involve endangered or protected species.

## Results

### Behavioural Consistency

**Figure 2:**
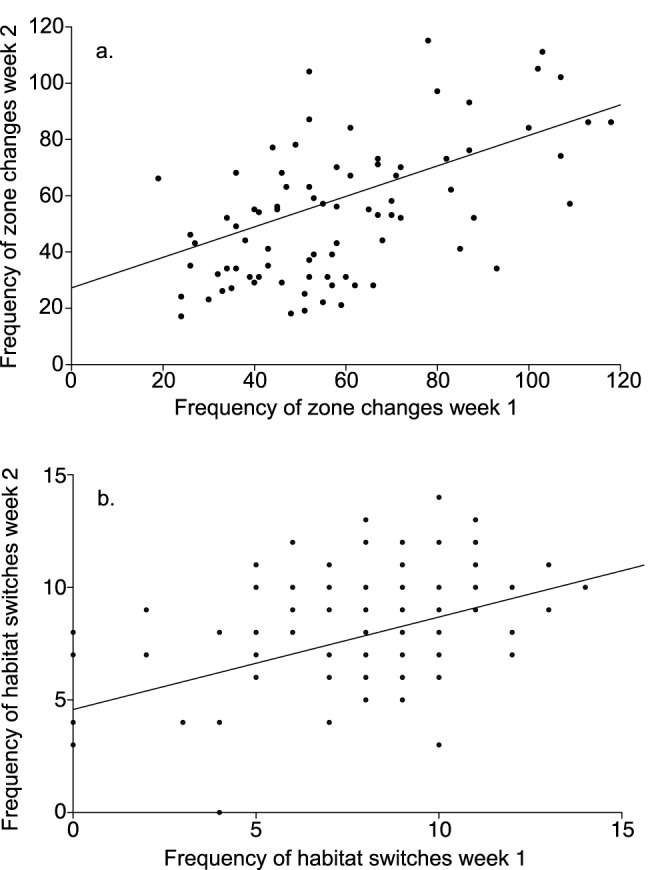
Individual consistency in (a) activity levels and (b) exploration in prawns.

At a population level, a number of behaviours showed consistency over time. Activity was significantly consistent over time (P<0.001, q<0.001: Fig. 2a) as was exploratory behaviour (P<0.001; q<0.001: Fig. 2b). There was a marginally significant trend towards fright response (boldness) being consistent over time (P = 0.04; q = 0.07). Neither habitat preference nor shoaling tendency were consistent over time (P>0.05, q>0.05).

### Behavioural Syndromes

**Figure 3:**
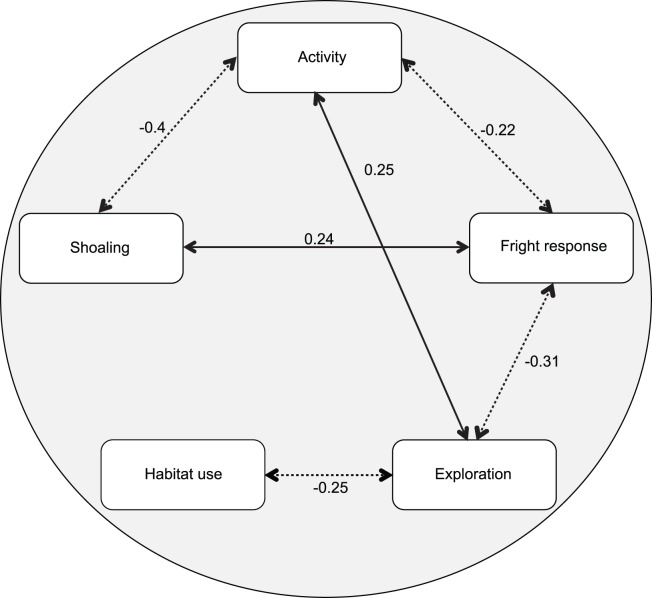
A behavioural syndrome in rock pool prawns. Arrows interlinking behaviours indicate significant relationships, with solid lines representing positive correlations and dotted lines for negative correlations. The correlation coefficient is also shown.

We found evidence of a complex behavioural syndrome at a population level as a number of behaviours correlated with one another in both week 1 and week 2 (week 1 shown in Fig. 3). In week 1 we found significant positive relationships between activity and exploration (P = 0.018, rho = 0.25, q = 0.05), fright response and shoaling tendency (P = 0.029, rho = 0.24, q = 0.058), and significant negative relationships between activity and shoaling tendency (P<0.001, rho = −0.4, q = 0.001), exploration and habitat fidelity (P = 0.017, rho = −0.25, q = 0.05), and exploration and fright response (P = 0.005, rho = −0.31, q = 0.024). We found a marginally significant negative relationship between activity and fright response (P = 0.049, rho = −0.22, q = 0.08). All other pairwise comparisons of behaviours showed no significant relationships (P>0.05, q>0.05).

In week 2 similar patterns of correlated traits were evident: there was a positive relationship between activity and exploration (P<0.001, rho = 0.37, q = 0.002) and a negative relationship between exploratory behaviour and fright response (P<0.001, rho = −0.4, q = 0.002). All other pairwise comparisons of behaviours showed no significant relationships (P>0.05, q>0.05).

**Figure 4:**
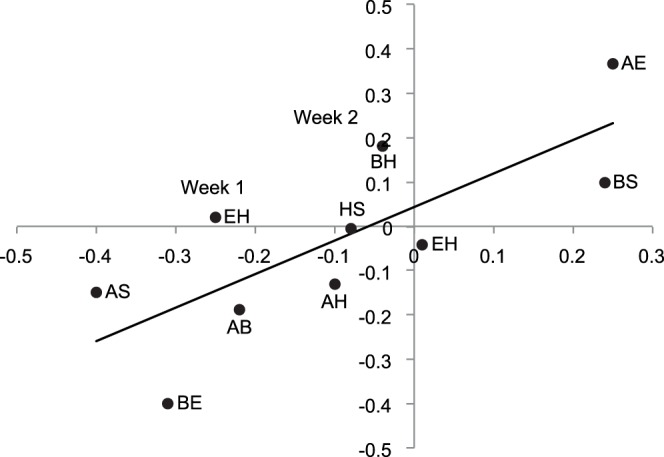
Behavioural syndrome structure is consistent over time in rock pool prawns. Each point represents the correlation coefficient between two traits for week one and week two. Trait combinations are indicated using the following notation: A = activity, B = boldness, E = exploration, H = habitat preference, S = shoaling tendency.

We found that behavioural syndrome structure (characterised as correlation coefficients between behavioural trait combinations) was significantly positively correlated over time, showing that the population behavioural syndrome was consistent over time in its structure (Mantel test 1,000,000 permutations: P = 0.049, rho = 0.77: Fig. 4).

### Sex Differences in Behaviour, Consistency and Syndrome Structure

**Figure 5:**
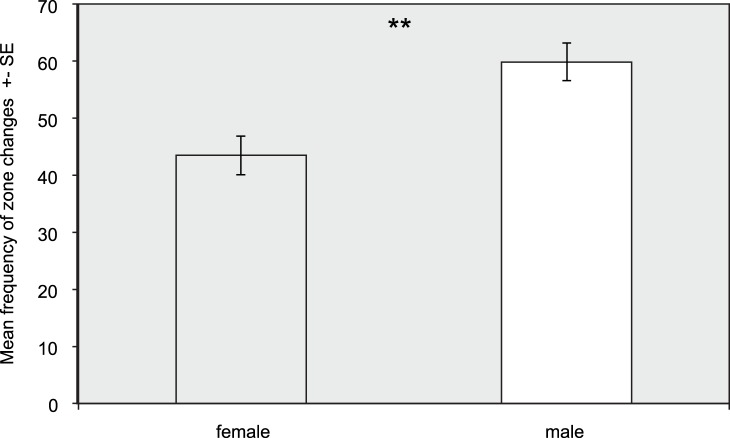
Sex differences in mean activity: males are significantly more active than females.

**Figure 6:**
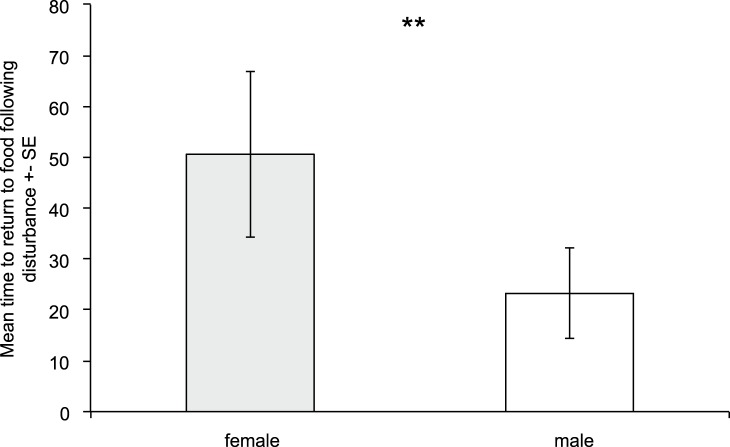
Sex differences in boldness: males are significantly bolder than females.

We found evidence for sex differences in behaviour between male and female prawns. Males were significantly more active (P = 0.002; q = 0.01: Fig. 5) and bolder (P = 0.009; q = 0.04: Fig. 6) than females in week 1. There were no differences in shoaling tendency, exploration or habitat preference. No sex differences in behaviour were evident in the second week. Males and females showed similar patterns of behavioural consistency to each other and the general population, with males being significantly consistent in activity (P = 0.003; q = 0.009) and exploratory behaviour (P = 0.002; q = 0.008). Females were consistent in activity alone (P<0.001; q<0.001). Using a Mantel test, we compared male and female syndrome structure for week 1, finding no correlation in syndrome structure between sexes (Mantel test, 1,000,000 permutations: P = 0.15, rho = 0.41).

## Discussion

Our study revealed that both individual behaviours and population-level behavioural syndrome structure are consistent in our study organism, the rock pool prawn. Individual prawns showed consistency in their activity patterns and exploratory behaviour over time, and showed patterns of inter-behavioural correlations indicating the presence of a complex behavioural syndrome in this species. Furthermore, we show that population syndrome structure is consistent over a period of 2 weeks. We also investigated sex differences in behaviour and personality, and found that males and females differ in behavioural expression in a number of behaviours, and also in behavioural syndrome structure.

Consistency in behavioural traits has now been documented in all major animal groups, and consistent individual differences commonly explain >30% of phenotypic variance within populations [38]. What shapes patterns of individual consistency is a major question in behavioural biology. Here we show that in the rock pool prawn, activity and exploratory behaviour are consistent over time, with a tendency for boldness also being consistent over time. Furthermore these behaviours were correlated as part of a behavioural syndrome in this population. We find relationships between activity, shoaling, fright response (i.e. boldness), exploration and habitat use. Individuals that exhibit high levels of activity are also highly exploratory, recover rapidly from a fright stimulus (i.e. are bold), and have a low propensity to form groups. These kinds of behavioural correlations have been widely documented across a range of taxa (e.g. 18,19). For example, bold sticklebacks shoal less than shy sticklebacks (12). Being in a group often has an anti-predatory function [39], and therefore shoaling is often considered to be a risk-averse strategy. Rock pool prawn are also thought to form groups to minimise the risk of predation [34], and hence having a low shoaling tendency may be a risk-prone behaviour in the wild. Furthermore, activity, exploration and boldness are often linked. Moving individuals are more conspicuous than stationary animals [40] and hence having a high activity level may also increase predation vulnerability. It is also possible that high activity involves an increased encounter rate with predators, which is likely to also increase the likelihood of mortality from a predator. In a similar way exploring new habitats is potentially dangerous. Finally, individuals with a high exploration score have a higher risk of predation due to their spending more time in the open habitat [41]. Exploration and activity were positively correlated in our study, and both were consistent over time. It is possible that high exploration scores are driven purely by high activity, as we report no habitat fidelity in prawns from our experiment. Hence what we refer to as ‘exploration’ may simply be an additional measure of activity in a different habitat setting. A challenge in many studies of behavioural syndromes is designing trials, which quantify a single trait in the absence of potentially related traits, especially in the case of behaviours which involve movement, such as exploration and activity.

We also analysed syndrome consistency across time, and found a strong relationship between syndrome structure in week one and week two. Few studies have considered the stability of behavioural syndrome structure over time, and hence our study is one of the first to report that syndromes (i.e. relationships between behaviours) in addition to single behaviours can also be consistent. A notable exception to this is recent work on hermit crabs *Pagurus bernhardus* [42] which showed that syndrome structure is consistent in that species. A sample of hermit crabs tested under different ecological conditions (low versus high perceived predation risk) displayed comparable behavioural syndrome structures across the two situations. A recent study investigating behavioural syndromes in brown trout *Salmo trutta* in the wild showed that a syndrome between average swimming velocity in different contexts emerged following a two-month period [21]. Further analyses suggested that the emergence of the syndrome was driven by a combination of natural selection and phenotypic plasticity, which supports data from experimental studies on stickleback *Gasterosteus aculeatus* [20]. How widespread syndrome stability is across taxa remains to be seen. That behavioural syndromes show strong stability may be indicative of the costs of behavioural plasticity outweighing the benefits of behavioural consistency. Interestingly syndrome structure was consistent over time despite that not all behavioural traits within the syndrome were individually consistent (e.g. shoaling behaviour was not stable over time).

Finally we report sex differences in behaviour and syndrome structure, but not consistency for rock pool prawns. Males were significantly more active and bolder than females in week one. This may be due to differences between males and females in the costs and benefits of expressing different degrees of behaviours. In this species there is sexual dimorphism for size, with females being larger than males [31]. Hence potentially females have a higher predation vulnerability than males, which means that there may be a greater cost to activity, which increases conspicuity to predators [40]. In addition, there is also often a skewed sex ratio for this species, with more males than females in natural populations [31]. Male-male competition for mating is low in rock pool prawns [43] and therefore there may be a potential benefit in high activity for males in terms of increasing the probability of encountering a receptive female and mating with her. Similarly boldness may be linked to increased reproductive success, potentially via mate choice, a preference for bold males has been documented in e.g. guppies [44]. Finally, males and females have different lifespans, with females living for twice as long as males [45]. Hence sex may influence an individual’s residual reproductive value, i.e. expected future fitness, which theoretical models suggest may be important in driving the evolution of personality [46]. As females live longer, theory would predict that they should be more risk-averse than males, as we show in this report. Future work could test some of the mechanisms we propose here to explain behavioural differences between the sexes. We found no significant relationship between syndrome structure for the sexes, which is suggestive of differences in syndrome structure. However, we are cautious in interpreting these results, as a lack of a significant relationship does not necessarily mean the absence of one. Our power was quite low due to sample size (i.e. number of behavioural traits quantified), and the effect size of the analysis was quite high (rho = 0.41). Qualitative inspection of potential differences in syndrome structure between males and females highlights differences in relationship direction between boldness-habitat preference and also shoaling tendency-habitat correlations. More work is clearly needed to further investigate sex differences in syndrome structure across a range of species.

To conclude, we report the presence of behavioural syndromes and individual consistency in the rock pool prawn, and in addition show that syndrome structure is consistent over time, and report sex differences in behaviour in this species. As the field of animal personality research matures and more data is made available on behavioural syndrome structure from across a range of species and populations, further work investigating inter- and intraspecific variation in syndrome structure and the causes of this variation becomes possible. These kinds of analyses would shed further light on to the evolutionary basis of personality variation in animals.
